# The effects of laser acupuncture on metabolic syndrome in obese postmenopausal women: a randomized controlled study

**DOI:** 10.1007/s10103-024-04158-0

**Published:** 2024-08-14

**Authors:** Wafaa M. Kamal, Ahmed M. Maged, Suzi AbdelAziz, Safaa I. Mahmoud, Reham A. Mohsen

**Affiliations:** 1 Departments of Physical Therapy for Woman’s Health, Faculty of Physical Therapy, Benha University, Benha, Egypt; 2 Department of Obstetrics and Gynecology, Kasr Al-Ainy Hospital, Cairo University, Cairo, Egypt

**Keywords:** Metabolic syndrome, Post-menopause, Obesity, Laser acupuncture

## Abstract

To study the laser acupuncture (LA) effects on postmenopausal obese women’s metabolic syndrome. Randomized controlled trial. Benha university hospital. Thirty postmenopausal women were randomized into two equal groups. Group A received a diet regimen and Group B received LA treatment for 30 min three times a week for two months beside the diet regimen. Included weight (W), body mass index (BMI), waist (WC), hip (HC), waist-hip ratio (WHR), systolic blood pressure (SBP), diastolic blood pressure (DBP), serum level of total cholesterol (TC), triglycerides (TG), high density lipoprotein (HDL), low density lipoprotein (LDL), fasting blood glucose (FBG), fasting blood insulin (FBI), homeostatic model assessment-insulin resistance (HOMA-IR) before and after the end of treatment. The study’s findings showed that both groups experienced a highly statistically significant decrease in the post-testing mean value of W, BMI, WC, HC, WHR, SBP, DBP, TC, TG, LDL, FBG, FBI, and HOMA-IR, while both groups experienced a significant increase in the post-treatment mean value of HDL (p 0.0001). The posttreatment SBP, DBP, TC, TG, LDL, FBS, FBI and insulin resistance were significantly lower while HDL was significantly higher in women who received combined LA and diet regimen compared to those who received dietary regimen only. LA beside the nutritional intervention is a physical therapy technique that may be used safely, easily, and effectively to minimize metabolic syndrome features during menopause.

## Introduction

Metabolic syndrome (MetS) is a pathophysiological condition that includes, dysglycemia, increased blood pressure, dyslipidemia and central obesity. All of which consider an increased risk of diabetes and cardiovascular diseases [1]. MetS diagnosed by presence of at least three of the following: more than 5.6 mmol/L (100 mg/dl) of blood sugar, males with HDL cholesterol below 1.0 mmol/L (40 mg/dl), and women with HDL cholesterol below 1.3 mmol/L (50 mg/dl), Waist > 102 cm for males or > 88 cm for women, blood triglycerides > 1.7 mmol/L (150 mg/dl), or Blood pressure > 130/85 mmHg [2].

Postmenopausal women are more likely than premenopausal women to have MetS [3, 4]. Numerous earlier investigations found that the incidence of MetS ranged from 3.1 to 22.9 per 1,000 women who were 40 years of age or older [5–7]. So, Perimenopausal period may be an excellent opportunity for preventative treatment to minimize metabolic risk factors and enhance health [8].

Drop in estrogens across menopause, may alter lipid and carbohydrates metabolism, trigger accumulation of visceral fat and reduce energy expenditure [9]. Hormonal changes after menopause, such as low level of plasma estrogen and with high levels of Follicle Stimulating Hormone (FSH) and Luteinizing Hormone (LH), have a significant effect on the metabolism of plasma lipid and lipoprotein, resulting in ultimate cardiac disorders. As, reduction in estrogen activity promote fat formation by increasing adipocyte size and number [10].

The synthesis of estrogen is nearly entirely stopped when the number of ovarian follicles declines to low levels during menopause [11]. The connection between menopause and other illnesses is due to the broad distribution of estrogen receptors (ER) along several physiological systems. Important estrogen targets include certain crucial systems including the cardiovascular system and the central nervous system. Postmenopausal women who are more susceptible to disease associated with estrogen deficiency, such as heart disease, osteoporosis and dyslipidemia [12].

A healthy lifestyle, which includes eating well, helps reduce the increased risk of metabolic syndrome in women who are going through menopause [13]. The EAT-Lancet Commission has even recommended a universal reference healthy diet with high consumption of fruit and vegetables and reduced intake of meat and refined sugar as among the primary recommendations, with the beneficial impact of a healthy diet being a prominent message [14]. In this regard, it has been suggested that the Mediterranean diet (MedDiet) is a good alternative [15], which takes care of women’s health concerns throughout and after the menopausal transition [16]. The MedDiet is a collection of suggestions that are represented in the food pyramid rather than a rigidly set diet [17].

The main suggested intervention for lowering the chances of getting MetS is proper diet [18]. The importance of nutrition in regulating the metabolic abnormalities linked to menopause has lately attracted more attention [19, 20].

In laser acupuncture (LA), acupuncture points are stimulated using lasers. At 35–40 mW of power, this type of acupuncture can produce chemicals including histamine, bradykinin, and adenosine triphosphate, which depolarize acupuncture sites. The brain receives this activity through afferent fibers, which in turn activates the limbic and hypothalamic systems. The hypothalamic center that controls appetite can be stimulated. Additionally, acupuncture causes the release of endorphins and can reduce hunger by reducing stress and despair. Adrenocorticotropin-releasing hormone (ACTH) will be released as a result of regulation in the hypothalamus. The liver’s adenylate cyclase enzyme may be increased by ACTH activation, which will subsequently activate the phosphorylation pathway that starts lipolysis and lowers triglyceride levels. Serotonin, which is produced by acupuncture, can also produces pro-opiomelanocortine (POMC) to reduce human appetite [21].

Menopausal symptoms and other gynecological disorders can be treated using laser acupuncture, a type of phototherapy, which is advised as a simple, non-invasive alternative to metal needles for stimulating acupuncture or musculoskeletal trigger points [22]. To distinguish it from broader laser therapeutic applications, it is commonly referred to as “laser acupuncture”.

The theoretical mechanism behind the effect of laser acupuncture is the process through which mitochondrial chromophores, particularly cytochrome c oxidase and photoreceptors in the plasma membrane of cells, absorb red and near-infrared light. This results in internal energy conversion and excitation of electrons at a lower energy level. It is hypothesized that light energy absorption causes inhibitory nitric oxide to be photo-dissociated from cytochrome c oxidase, improving enzyme activity, electron transport, mitochondrial respiration, and ATP generation [23].

Therefore, the goal of this study was to determine how Laser Acupuncture affected metabolic syndrome features in obese postmenopausal women.

## Subjects

A physician had recommended 30 post-menopausal women who had been diagnosed with the MetS. These women were recruited from outpatients clinics at Benha university hospital between December 22, 2022 and July 18,2023. The Adult Treatment Panel III (NCEP-ATP III) of the National Cholesterol Education Program was used to diagnose MetS. The post-menopausal women met three or more of the following criteria: a waist circumference of more than 88 cm, high TG (> 150 mg/dl or 1.65 mmol/L), low HDL-C (> 50 mg/dl or 1.30 mmol/L), and elevated fasting blood glucose (> 110 mg/dl or 6.1 mmol/L). Obesity was reported in all post-menopausal women (BMI from 30 to 34.9 Kg/m^2^) and their age ranged from 55 to 65 years. all women were had prehypertension or hypertension stage I as diagnosed by physician using the World Health Organization-International Society of Hypertension Guidelines for the Management of Hypertension [24–27], Women with a 159 mm Hg or higher systolic and 99 mm Hg or higher diastolic blood pressure, diabetic disease, mental or psychological disorders, hypothyroidism, ischemic heart disease (IHD), musculoskeletal disorders or who had taken hormone replacement therapy (HRT) were excluded from the study. The Faculty of Physical Therapy at Cairo University’s ethical review board gave the study approval number of (P.T.REC/012/004041). All participants have signed an informed written consent after describing the study’s purpose, methodology and advantages. The trial was prospectively registered at clinical trial registry with NCT05651451 number. The participants were divided using computer-generated random numbers equally into two groups; there were fifteen postmenopausal women given only an 8-week food plan in Group (A), and fifteen postmenopausal women in Group (B), followed the same food plan. Additionally, they received 24 sessions of 30-minute LA treatments over the course of eight weeks (3 sessions/week).

Based on a pilot investigation, the sample size was estimated using the difference between the two study groups’ post-treatment mean values, with an effect size of 0.95. A sample size of 15 patients per group would be needed if the significance level 0.05 and the test’s 80% power were assumed.

## Assessment procedures

### Anthropometric assessment

Every post-menopausal lady who was dressed in light clothing and was shoeless had her height and weight taken. BMI was then computed by dividing weight (kg) by height squared (m2). The waist and hip circumferences were measured preceding and following the 8-weeks treatment by the same therapist. At the end of normal expiration, the therapist measured the waist circumference from the narrowest point between the lower border of the rib cage and the iliac crest; she measured the hip circumference from the broadest section of the hip. The waist-hip ratio was then determined by dividing the waist circumference by the hip circumference.

### Blood pressure assessment

The auscultatory method of measuring blood pressure was used with a sphygmomanometer properly calibrated and validated. Blood pressure was evaluated preceding and following the 8-weeks treatment. Following an overnight fast and a minimum of 24 h of abstinence from caffeinated beverages, alcohol, and strenuous or prolonged physical activity, this procedure was carried out in a calm, temperature-controlled room (23 °C) using suggested techniques and categories from the World Health Organization-International Society of Hypertension Guidelines for the Management of Hypertension.

### Biochemical assessment

After fasting for 9–12 h, blood samples were collected from all postmenopausal women in clean tubes containing a few milligrams of K2EDTA. Blood samples were centrifuged to remove plasma, which was then frozen at -20° until examination. Plasma concentrations of TC, HDL, LDL, TG, FBG, FBI, and HOMA-IR were measured before and after treatment program. HOMA-IR was calculated using the formula of: fasting glucose (mg/dL) X fasting insulin (mU/L) / 405.

## Treatment procedures

### Dietary regimen

For 8 weeks, all post-menopausal women adhered to an energy-restricted diet. First, the Harris-Benedict equation was multiplied by 1.375 to determine the required daily calorie intake. The daily calorie requirement was then restricted by 1000 kcal/day to set the daily energy consumption limit.

Each post-menopausal woman followed DASH diet which: Low in sweets, fat, red meat, and beverages with added sugar, emphasize fish, nuts, whole grains, fruit and vegetables.

Each post-menopausal lady received a pamphlet with a food database, along with information on the items’ energy and macronutrient contents. Each lady was given full reign to choose her own foods, along with instructions on how to organize her meals in order to stay within the allotted number of calories and macronutrients. The therapist conducted weekly interviews with all women and instructed them to keep 3-day nutrition logs. The therapist went through these statistics to make sure the daily total of calories was within the previously determined range.

### Laser acupuncture

Low-level laser treatment (LLLT) (EME srl, Italy; model Lis 1050, SN: EM 13481014) was used. With a wave length of 905 nm, an average radiant power output of 12 mW, a pulse frequency of 5000 Hz, a pulse radiation duration of 100 ns, and an energy density of 2 J/cm2, each acupoint was exposed to radiation for 3 min. To prevent the beam from being scattered, the laser light was applied perpendicularly. The acupuncture sites listed below were chosen for laser irradiation:

- Cv4 (Guanyuan) is 3 cm below the umbilicus’s center.
- Cv9 (Shuifen) is 1 cm above the umbilicus’s center; and.
- Cv12 (Zhongwan) is 4 cm above the umbilicus’s center.
- St36 (Zusanli) (one finger width lateral from the anterior crest of the tibia).
- St25 (Tianshu) (2 cm lateral to the midline of the umbilicus).
- LI4 (Hegu) (on the highest point of the muscle when the thumb and index fingers are pulled together).
- Sp6 (Sanyinjiao) (3 cm immediately above the tip of the medial malleolus).
- St40 (Fenglung) (8 cm superior to the apex of the external malleolus).
- LI11 (Quchi) (on the outside end of the crease on the elbow).
- And PC6 (Neiguan)(Three finger widths down from the wrist, between the two tendons on the inside forearm.

## Statistical procedures

Data analysis was performed using the Statistical Package for Social Sciences (SPSS) application (version 23 windows). Also, different variables were compared between groups by the help of unpaired t-test as regarding data which was normally distributed. The assumption of normality, the homogeneity of variance, and the presence of extreme scores were checked on the data, this was required for the examination of difference’s parametric computations. All of the dependent variables had been normally distributed and did not violate the parametric assumption for the measured dependent variable, according to descriptive analysis utilizing histograms and the normal distribution curve. There was no discernible change when the homogeneity of covariance was tested as well. The paired t-test was used to compare pre- and post-assessments within the same group in pairs. With P value ≤ 0.05.

## Results

**Fig. 1:**
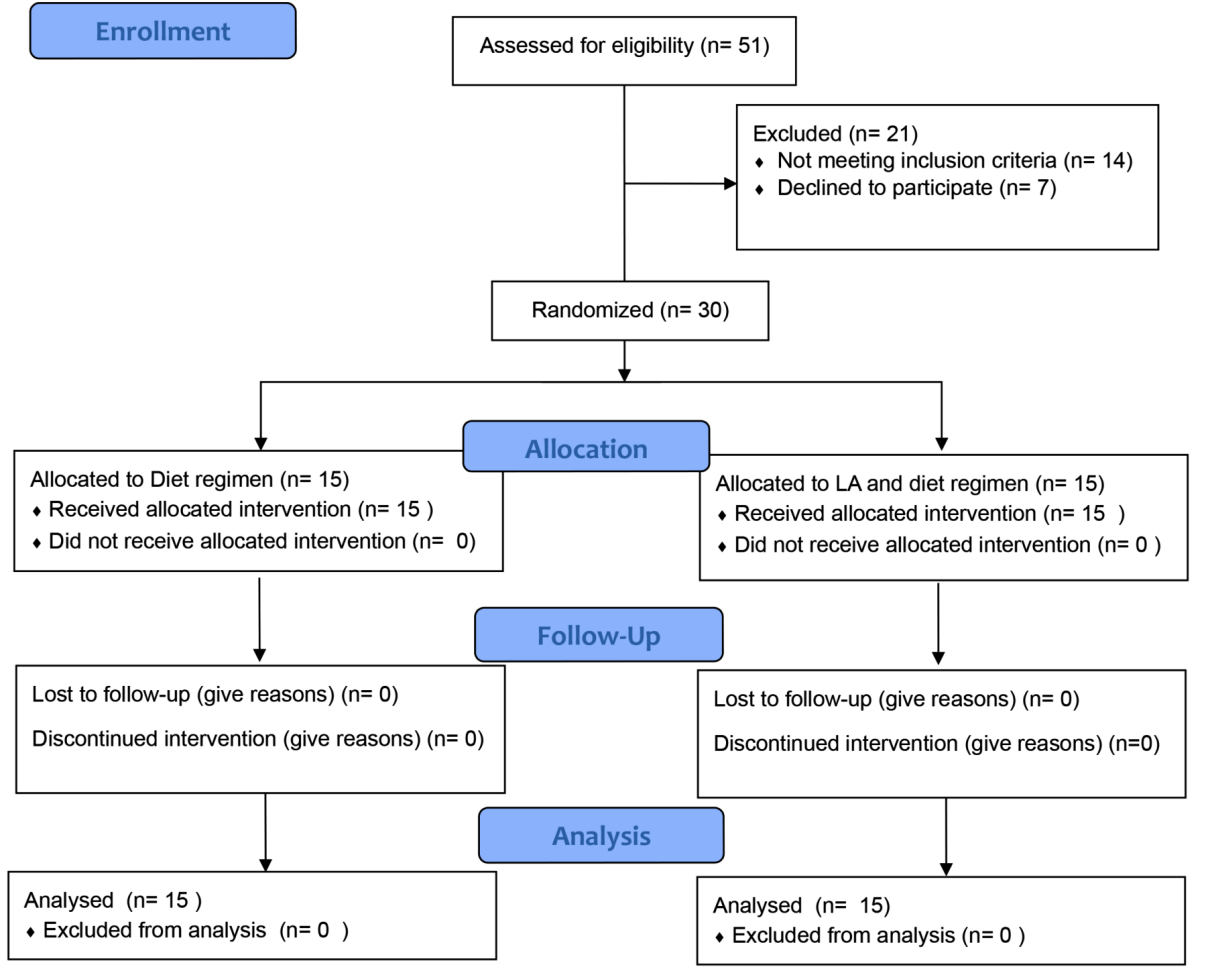
Consort flow chart

The consort flow chart of the study is shown in Fig. 1.

No significant difference was found between the 2 study groups regarding age, weight, height, body mass index or duration of menopause (Table 1).

**Table 1:** Physical characteristics of participants

	Group A (Diet group) (*n* = 15)	Group B (LA group) (*n* = 15)	unpaired t-test		95% CI
Age (years)	59.13 ± 2.88	59.00 ± 2.65	0.13	0.89	-1.93 to 2.20
Weight (Kg)	89.47 ± 4.00	90.27 ± 5.01	0.48	0.63	-4.19 to 2.59
Height (cm)	161.93 ± 4.18	163.00 ± 4.66	0.66	0.51	-4.38 to 2.25
BMI (Kg/m2)	34.17 ± 0.79	33.96 ± 0.96	0.47	0.63	-0.50 to 0.81
Duration of menopause (years)	8.07 ± 2.55	7.40 ± 2.44	0.73	0.47	-1.20 to 2.53

BMI Body mass index; LA Laser acupuncture

Data are presented as mean ± SD

Regarding anthropometric parameters, there were no differences between the baseline weight, BMI, WC, HC or WHR. All the parameters showed significant differences between measurements in the pretreatment and posttreatment measures in both groups. Significant differences were detected between the posttreatment WC and WHR when comparing the 2 groups. This difference was not detected in W, BMI, or HC (Table 2).

**Table 2:** Comparison between anthropometric parameters in the two study groups

		Group A (Diet group) (*n* = 15)	Group B (LA group) (*n* = 15)	t-test	*P* value
Weight (W)kg	Baseline	89.47 ± 4.00	90.27 ± 5.01	0.48	0.63
	After treatment	79.40 ± 3.89	80.33 ± 5.02	0.57	0.57
	Changes	10.07	9.94		
	P value	0.0001	0.0001		
Body mass index (BMI) (kg/m2)	Baseline	34.12 ± 0.79	33.96 ± 0.96	0.47	0.64
	After treatment	30.04 ± 0.85	30.24 ± 1.46	0.46	0.65
	Changes	4.08	3.72		
	P value	0.0001	0.0001		
Waist circumference (WC) cm	Baseline	105.40 ± 5.32	106.07 ± 4.64	0.37	0.72
	After treatment	96.73 ± 5.34	92.80 ± 4.44	2.19	0.04 *
	Changes	8.67	13.27		
	P value	0.0001	0.0001		
Hip circumference (HC) cm	Baseline	115.27 ± 3.92	116.27 ± 3.81	0.71	0.48
	After treatment	109.20 ± 4.46	108.33 ± 2.55	0.65	0.52
	Changes	6.07	7.94		
	P value	0.0001	0.0001		
Waist-hip ratio (WHR)	Baseline	0.91 ± 0.03	0.91 ± 0.02	0.08	0.94
	After treatment	0.89 ± 0.03	0.86 ± 0.03	2.52	0.02 *
	Changes	0.02	0.05		
	P value	0.0001	0.0001		

Data are presented as mean ± SD

The posttreatment SBP, DBP, TC, TG, HDL, LDL, FBS, FBI, HOMA-IR showed significant differences from pretreatment measures in both groups (Table 3). The posttreatment SBP, DBP, TC, TG, LDL, FBS, FBI and insulin resistance were significantly lower while HDL was significantly higher in women who received combined LA and diet regimen compared to those who received dietary regimen only (Table 3).

**Table 3:** Comparison between metabolic parameters in the two study groups

		Group A (Diet group) (*n* = 15)	Group B (LA group) (*n* = 15)	t-test	*P* value
Systolic blood pressure (SBP)	Baseline	149.00 ± 5.07	148.33 ± 5.56	0.34	0.73
	After treatment	143.67 ± 5.16	135.67 ± 4.95	4.33	0.0002
	Changes	5.33	12.67		
	P value	0.0021	0.0001		
Diastolic blood pressure (DBP)	Baseline	92.67 ± 2.58	93.33 ± 2.44	0.72	0.47
	After treatment	87.33 ± 3.20	78.00 ± 3.68	7.41	0.0001
	Changes	5.33	15.33		
	P value	0.0001	0.0001		
Total cholesterol ( mg/dL)	Baseline	279.87 ± 18.54	283.40 ± 11.55	0.62	0.53
	After treatment	262.53 ± 17.05	234.33 ± 8.66	5.71	0.0001
	Changes	17.33	49.07		
	P value	0.0001	0.0001		
Triglycerides (mg/dL)	Baseline	171.93 ± 15.30	178.93 ± 13.76	1.31	0.198
	After treatment	163.47 ± 15.61	141.60 ± 8.65	4.74	0.0001
	Changes	8.47	37.33		
	P value	0.0001	0.0001		
High density lipoprotein (mg/dL)	Baseline	48.73 ± 4.11	47.47 ± 4.79	0.77	0.44
	After treatment	51.87 ± 3.94	55.87 ± 2.8	3.20	0.0034
	Changes	-3.13	-8.40		
	P value	0.0001	0.0001		
Low density lipoprotein (mg/dL)	Baseline	210.20 ± 19.07	208.47 ± 17.07	0.26	0.79
	After treatment	191.87 ± 13.23	163.93 ± 11.35	6.21	0.0001
	Changes	18.33	44.54		
	P value	0.0001	0.0001		
Fasting blood glucose (FBG) (mmol/l)	Baseline	9.29 ± 1.76	8.98 ± 2.06	0.45	0.66
	After treatment	7.66 ± 1.39	7.34 ± 1.74	0.54	0.59
	Changes	1.63	1.64		
	P value	0.0001	0.0001		
Fasting blood insulin (FBI) (µIU/ml)	Baseline	11.46 ± 1.28	12.13 ± 2.18	1.03	0.31
	After treatment	9.62 ± 0.98	8.59 ± 1.63	2.10	0.04
	Changes	1.84	3.54		
	P value	0.0001	0.0001		
Insulin resistance (HOMA-IR)	Baseline	4.42 ± 0.86	4.85 ± 0.64	1.56	0.13
	After treatment	3.00 ± 0.78	2.83 ± 0.79	0.60	0.55
	Changes	1.42	2.02		
	P value	0.0001	0.0001		

Data are presented as mean ± SD

## Discussion

Numerous metabolic components are adversely impacted by menopause. These detrimental consequences may be minimized by eating a balanced diet and following a healthy lifestyle.

The results of this study revealed a highly statistically significant decrease in the post-testing mean values of W, BMI, WC, HC, WHR, SBP, DBP, TC, TG, LDL, FBG, FBI, and HOMA-IR in both groups (A and B) (p 0.0001), while there was a significant increase in the post-treatment mean value of HDL in both groups (A and B) (p 0.0001).

These results are supported by **Vasei et al.** [24], **Guo et al.** [25], **Lari et al.** [26] & **Soltani et al.** [27], they showed that the DASH diet could reduce weight, WC, TC, TG and LDL in obese people with metabolic syndrome, in addition to systolic and diastolic blood pressure. Also, **Campbell et al.** [28] & **Rooholahzadegan et al.** [29], found that DASH diet results in a considerable increase in sensitivity of insulin and associated indicators.

These results are also consistent to **Al-Solaimanet al.** [30], **Ndanuko et al.** [31] **& Rifaiet et al.** [32] who discovered that the DASH content of fibers, antioxidants, flavonoids, calcium, potassium and magnesium, explain their positive effects on endothelial and vascular functions. Calcium modifies peripheral vascular resistance via regulating the contractility of smooth muscles in the vascular system which in turn regulates blood pressure. Additionally, through interacting with the calcium receptor, extracellular ionized calcium reduces renin secretion.

In comparison to the control group, the acupuncture group had a significant decrease in waist circumference (WC) and waist hip ratio (WHR). This is consistent with **El-Mekawy et al.** [33] who revealed that diet-exercise programs with the addition of laser acupuncture resulted in greater advantages for lowering waist and hip circumference, and **Shen et al.** [34], who concluded that acupuncture could successfully lower WC in people with PCOS who have abdominal fat.

Acupuncture can reduce abdominal obesity and shield against metabolic diseases through regulating gut flora [35], or through its control of hypothalamus’ autophagy and its impact on TSC1-mTOR. [36], or through regulating intestinal motility, food intake, and secretion of LEP and CCK [37].

The findings of our investigation revealed no statistically significant variations in weight or BMI between the two groups. This finding matches **El-Mekawy et al.** [33] and **Zhang et al.** [38], both found non-significant difference in the weight after the laser acupuncture treatment. Also, **Avci et al.** [39] **as well as**,** Caruso-Davis et al.** [40] suggested that low-level laser treatment seems to be a nonsurgical method for mobilizing local fat to reshape body without causing weight loss. The fat loss was likely caused by the laser producing transient holes through adipocyte’s membrane where triglycerides released.

Contrary to our results, **Sebayang et al.** [21], **Hu et al.** [41] **& Hung et al.** [42], whose findings demonstrated considerable differences in body weight and BMI values in laser acupuncture group against control group (*P* < 0.0001). The longer duration of those studies might be the cause of this discrepancy. Additionally, it included obese participants whose mean age was younger than that of the current study. So, aging results in rise in fatty tissues and skeletal muscle loss, which lowers BMR and fat oxidation [43].

The acupuncture group had considerably lower systolic and diastolic blood pressure (SBP&DBP) than the control group. These outcomes matched those of **Zhang et al.**, [38], **Sudhakaran.**, [44] **&Çevik&İşeri**, [45] who believed that stimulating nitric oxide synthase by the low laser therapy of acupoints LI-11, LI-4, and ST-36 lowers blood pressure. Additionally, they discovered that electro-acupuncture raised the peri-arteriolar nitric oxide. Additionally, in accordance with **Jiang et al.**, [46] who concluded that the skin surface can control blood flow, blood pressure, and blood nitric oxide levels. This is because acupuncture may alter the perfusion of interior organs by microcirculation.

Additionally, **Man et al.** [47] came to the conclusion that the antihypertensive mechanism of acupuncture is impacted by genes, metabolism, vascularity, brain activity, oxidation response, and renin-angiotensin-aldosterone system (RAAS).

The acupuncture group had a considerably higher reduction in TC, TG, LDL, and a rise in HDL levels than control group. These results are in line with **El-Mekawy et al.** [33] who concluded that TC concentration is reduced when a mix of laser acupuncture plus diet-exercise plan is employed. Also these findings concurred with **Li et al.** [48] who confirmed that acupuncture can reduce TG, TC, and LDL levels while raising HDL levels to control lipid metabolism. So, it can prevent hyperlipidemia and safeguard vascular endothelial function.

In the current investigation, the laser acupuncture group demonstrated a significantly higher reduction in insulin levels than control group, indicates that insulin sensitivity has improved and insulin resistance has reduced. This finding agreed with **Li et al.** [48] who proved that laser acupuncture exhibited beneficial impact on insulin sensitivity in diabetic rats, and could be used to treat type 2 diabetes.

Also, **El-Shamy et al.** [49] as well as, **Shen et al.**, [34] proved that acupuncture could successfully lower WC, fasting insulin and HOMA-IR in obese women, and they confirmed that the laser acupuncture is much more potent than diet plus exercise. Also, the systematic review of **Zheng et al.** [50], **as well as**,** Liu et al.** [51] confirmed that in terms of enhancing insulin sensitivity and glucose metabolism, acupuncture is generally safe and effective.

The exact way laser acupuncture alters insulin sensitivity is still a mystery. The increase in insulin sensitivity, however, may be explained by a variety of physiological theories. **An et al.** [52] suggested that acupuncture can stimulate movement of the intestines to enhance the bacteria in the gut, so lowering blood glucose level by lowering the amount of inflammatory processes through the signal of IKKβ/NF-κB-JNK-IRS-1-AKT pathway. While **Lu et al.** [53] suggested that acupuncture could promote insulin sensitivity and diminish inflammatory reactions by blocking the pathway of TLR4/NF-κB in liver. Also, **Yang et al.** [54] concluded that acupuncture is more effective in improving lipid metabolism in obese mice by lowering hepatic TLR4/NF-B p65 signaling.

The combined action of the laser beam and acupuncture may account for all of these advancements in the laser acupuncture group. Acupuncture may reduce obesity through a variety of ways, as downregulation and decreased synthesis of AgRP and NPY as well as, upregulation and increased synthesis of MSH and POMC in the ARC, the decreased activity of LHA neurons and the increased activity of VMH neurons, a rise in CCK, a reduction in leptin and insulin and increased its sensitivity [55]. Additionally, adipocytes’ mitochondria are stimulated by low-level laser treatment, which results in an increase in the production of (ATP) and (cAMP). Also, activate lipase enzyme which breaks down triglycerides into glycerols and free fatty acids that enter the extracellular space through pores generated in the cell [56].

Our Findings point to a potential function for laser acupuncture in enhancing lipid metabolism, lowering blood pressure and improving insulin sensitivity and quality of life of post-menopausal women. Although laser acupuncture helps obese post-menopausal women with their MetS symptoms, the underlying reasons are still unclear, which calls for more investigation.

Despite the fact that the current study provides objective data, numerous limitations were noted, including the study’s small sample size, lack of follow-up, and assessment of the long-term effects of laser acupuncture. Additionally, the DASH diet regimen and stage I hypertension were the only selection criterion. Therefore, more research is required to examine the impact of laser acupuncture therapy on various phases of hypertension together with diverse food regimens in order to improve the generalizability of the findings.

## Conclusion

Combining laser acupuncture with a diet intervention could reduce fasting insulin levels, reduces waist circumferences, and reduces plasma lipids. These results imply that laser acupuncture may help post-menopausal women with MetS improve their abdomen obesity, lipid profile, blood pressure and glucose-insulin balance.
